# Donor-Derived Fibrin Matrix as an Adjunct to Microsurgical Recurrent Laryngeal Nerve Repair: An Experimental Rat Study

**DOI:** 10.3390/biomedicines14102276

**Published:** 2026-10-08

**Authors:** Alper Varman, Naile Kökbudak, Ömer Kişi, Celalettin Vatansev

**Affiliations:** 1 General Surgery Department, Faculty of Medicine, Necmettin Erbakan University, 42090 Konya, Türkiye; 2 Pathology Department, Faculty of Medicine, Necmettin Erbakan University, 42090 Konya, Türkiye

**Keywords:** recurrent laryngeal nerve, fibrin matrix, nerve repair, microsurgery, vocal fold, muscle atrophy, rat model

## Abstract

**Background/Objectives:** Recurrent laryngeal nerve (RLN) transection may cause persistent vocal fold dysfunction despite microsurgical reconstruction. Biological scaffolds may support nerve healing and preserve target muscle. This study evaluated a donor-derived fibrin matrix as an adjunct to primary microsurgical RLN repair in rats. **Methods:** 27 female Wistar albino rats were assigned to three groups (*n* = 9 each): RLN transection without repair, primary end-to-end microsurgical repair, and identical repair supplemented with donor-derived fibrin matrix. Vocal fold mobility was assessed by laryngoscopy after 3 months. Vocal fold muscle atrophy and histopathological changes at the RLN injury/repair site were evaluated. **Results:** Vocal fold mobility was observed in 0/9, 2/9, and 4/9 animals, respectively, without a significant between-group difference (*p* = 0.116). Vocal fold muscle atrophy differed significantly among groups (*p* < 0.001). Severe atrophy occurred in all animals in the transection group and in 4/9 after primary repair, whereas no severe atrophy occurred in the fibrin matrix group; 7/9 animals in this group had no atrophy. Significant group differences were also observed for inflammation (*p* = 0.003), edema (*p* = 0.008), fascicular organization disorder (*p* < 0.001), and foreign-body reaction (*p* < 0.001). Axon counts did not differ significantly (*p* = 0.085). **Conclusions:** Donor-derived fibrin matrix supplementation was associated with reduced vocal fold muscle atrophy and more favorable histopathological findings after microsurgical RLN repair. Although vocal fold mobility was numerically higher in the fibrin matrix group, the difference was not statistically significant; therefore, no definitive improvement in functional recovery can be concluded. These findings suggest a potential structural benefit of fibrin matrix supplementation and warrant further investigation in larger studies with more sensitive functional assessments.

## 1. Introduction

Recurrent laryngeal nerve (RLN) injury remains one of the most concerning complications of thyroid and parathyroid surgery. Although improvements in surgical technique, anatomical knowledge, and intraoperative nerve monitoring have contributed to safer surgery, temporary or permanent RLN dysfunction continues to occur in clinical practice [1,2]. Unilateral RLN injury may result in dysphonia, impaired swallowing, ineffective cough, and aspiration, whereas bilateral injury can cause significant airway compromise. Beyond these immediate consequences, persistent vocal fold dysfunction can substantially affect communication and quality of life [1,3]. Therefore, when the RLN is transected or intentionally sacrificed and both nerve ends are available, restoration of neural continuity is an important reconstructive goal.

Several techniques have been described for RLN reconstruction, including direct end-to-end anastomosis, free nerve grafting, ansa-cervicalis-to-RLN anastomosis, and, more recently, artificial nerve conduits [3,4]. When a tension-free repair is technically possible, direct microsurgical anastomosis represents the most straightforward approach. Clinical evidence suggests that immediate RLN reconstruction can improve phonatory outcomes and help preserve vocal fold tone and muscle bulk [1,3]. Nevertheless, anatomical continuity does not necessarily translate into normal vocal fold motion. Regenerating axons may undergo misdirection and inappropriate reinnervation of the laryngeal adductor and abductor muscles, resulting in synkinesis and incomplete functional recovery despite successful nerve regeneration [5]. This discrepancy between structural regeneration and functional recovery has prompted increasing interest in biological and tissue-engineering strategies that may provide a more favorable environment for regenerating axons.

Fibrin-based matrices are particularly attractive in this context because they can provide a biodegradable three-dimensional scaffold capable of supporting cellular migration and axonal extension during peripheral nerve healing [6]. More recent experimental and translational evidence has further supported the use of fibrin-based materials as adjuncts to peripheral nerve repair. A recent scoping review highlighted the potential of fibrin biopolymers used in association with neurorrhaphy to create a protective local microenvironment that may facilitate nerve and neuromuscular regeneration. In experimental peripheral nerve models, platelet-rich fibrin incorporated into bioabsorbable nerve conduits has been associated with increased axonal regeneration and improved sensory recovery, while fibrin glue used for nerve graft fixation has produced functional and histological outcomes broadly comparable to conventional microsurgical neurorrhaphy [7,8,9]. Importantly, recent RLN-specific studies using platelet-derived biological adjuncts have also demonstrated enhanced Schwann-cell activity, neural regeneration, electrophysiological recovery, and preservation of target muscle, supporting further investigation of blood-derived biological matrices in RLN repair [10,11].

However, evidence regarding the use of a donor-derived fibrin matrix specifically as an adjunct to primary microsurgical RLN anastomosis remains limited. Previous regenerative approaches have primarily focused on engineered nerve conduits, platelet-rich plasma, platelet-rich fibrin, or other biomaterial-based scaffolds, whereas the direct application of a donor-derived fibrin matrix around an already completed end-to-end RLN anastomosis has not been adequately investigated.

Among the available regenerative strategies, a donor-derived fibrin matrix offers several potential practical advantages as an adjunct to microsurgical RLN repair. Unlike nerve conduits, it does not replace or bridge the primary repair but can be applied directly around an already completed end-to-end anastomosis. In contrast to more complex engineered biomaterials, the matrix can be prepared from blood using a relatively simple centrifugation protocol and does not require a manufactured scaffold. Furthermore, its fibrin-rich structure may provide a temporary three-dimensional biological environment that supports cellular migration and axonal growth while retaining locally active blood-derived mediators. These characteristics make it a potentially attractive adjunct when anatomical continuity can already be restored by microsurgical repair. Although the present experimental model used donor-derived material, a clinically relevant extension of this approach could involve an autologous fibrin matrix prepared intraoperatively from the patient’s own blood. However, the present study should be regarded as a preclinical proof of concept and does not establish clinical efficacy or safety. Therefore, it remains unclear whether modifying the local biological microenvironment with such a matrix can improve nerve healing, preserve the target laryngeal muscle, or enhance functional recovery after RLN transection and repair. This represents a specific knowledge gap in the current literature.

Unlike engineered nerve conduits or commercially prepared fibrin sealants, a blood-derived fibrin matrix can be prepared using a relatively simple centrifugation protocol and applied directly to the repair site without replacing the primary microsurgical anastomosis. The novelty of the present study lies in evaluating this donor-derived fibrin matrix as a biological adjunct to a conventional primary end-to-end RLN repair, with simultaneous assessment of vocal fold mobility, target-muscle atrophy, and histopathological changes at the repair site. Mechanistically, the fibrin matrix was expected to provide a temporary three-dimensional scaffold around the repaired nerve, supporting cellular migration and axonal extension while stabilizing the local repair microenvironment; these effects could potentially improve the organization of regenerating fibers, preserve neuromuscular connectivity, and reduce denervation-related target-muscle atrophy. Therefore, the present study aimed to compare untreated RLN transection, primary microsurgical end-to-end repair, and primary repair supplemented with a donor-derived fibrin matrix in a rat model. Functional recovery was assessed by vocal fold mobility, while the effects of repair were further evaluated through vocal fold muscle atrophy and histopathological changes at the RLN repair site. We hypothesized that the supplementation of primary microsurgical repair with a donor-derived fibrin matrix would improve the local regenerative microenvironment, resulting in better preservation and organization of regenerating nerve tissue and target laryngeal muscle, with the potential to enhance functional recovery.

## 2. Materials and Methods

This experimental study was conducted at the Experimental Medicine Application and Research Center (KONÜDAM), Necmettin Erbakan University, Konya, Türkiye. An a priori sample size calculation was performed based on the primary functional outcome of vocal fold mobility at 3 months. Assuming expected mobility rates of 0%, 30%, and 70% in the transection, primary repair, and fibrin-matrix-supplemented repair groups, respectively, corresponding to a large overall effect size (Cohen’s w = 0.61), a total sample size of 27 animals (nine per group) was required to achieve 80% statistical power at a two-sided α level of 0.05. A total of 27 3-month-old female Wistar albino rats were included in the experimental groups (*n* = 9 per group), and one additional donor rat was used for preparation of the fibrin matrix, resulting in a total of 28 animals used in the study. All surgical procedures, postoperative follow-up, and animal care were performed at KONÜDAM. The experimental protocol was approved by the Necmettin Erbakan University KONÜDAM Local Animal Ethics Committee on 28 November 2024 (Decision No. 2024-113). Animals were maintained under standard laboratory conditions with access to standard laboratory chow and water. All efforts were made to minimize animal discomfort and the number of animals used. The study is reported in accordance with the ARRIVE 2.0 guidelines for animal research.

### 2.1. Experimental Design

At the beginning of the experiment, 28 female Wistar albino rats were available. Before allocation of the experimental animals, one rat was randomly selected to serve as the donor for fibrin matrix preparation. The remaining 27 rats were then randomly assigned to the three experimental groups (*n* = 9 per group) using a computer-generated randomization sequence. The donor animal was not included in any of the experimental groups.

Group 1 (transection group, *n* = 9): The left RLN was transected and left unrepaired.

Group 2 (primary repair group, *n* = 9): Following transection of the left RLN, primary end-to-end microsurgical anastomosis was performed using 8/0 Prolene sutures under 10× microscopic magnification.

Group 3 (fibrin matrix group, *n* = 9): Following left RLN transection, primary end-to-end microsurgical anastomosis was performed using the same technique as in Group 2, and a donor-derived fibrin matrix was subsequently applied over the anastomosis site.

The individual rat was considered the experimental unit. Before the surgical procedure, bilateral vocal fold mobility was confirmed by transoral laryngoscopic examination. Following RLN transection, loss of mobility of the ipsilateral vocal fold was confirmed laryngoscopically. Animals were then followed for 3 months, after which vocal fold mobility was reassessed and tissue specimens were obtained for histopathological examination.

Because of the nature of the surgical interventions, the operating researchers were not blinded to group allocation. For histopathological assessment, however, tissue samples were coded by group number, and the investigators performing the histopathological evaluation were blinded to the corresponding treatment allocation. All 27 experimental animals completed the 3-month follow-up and were included in the final analyses; no animals or experimental data were excluded.

### 2.2. Preparation of the Donor-Derived Fibrin Matrix

The fibrin matrix used in Group 3 was prepared from approximately 5 mL of fresh whole blood obtained by intracardiac sampling from a separate donor rat under general anesthesia. The blood was collected into a sterile tube without anticoagulant and immediately centrifuged at 400× *g* for 10 min using a platelet-rich fibrin (PRF)-based preparation protocol. Following centrifugation, the fibrin-rich clot formed between the lower erythrocyte fraction and the upper plasma fraction was separated under sterile conditions.

The resulting fibrin matrix was used immediately after preparation. For each animal in Group 3, a visually comparable portion of the matrix was placed directly over and around the completed microsurgical RLN anastomosis, in an amount sufficient to cover the repair site without causing compression of the nerve or surrounding tissues. The exact weight and dimensions of the individual fibrin portions were not prospectively measured.

The preparation method was based on the protocol described by Huang et al., in which blood collected without anticoagulant was centrifuged at 400× *g* for 10 min to obtain the intermediate fibrin clot [12].

### 2.3. Anesthesia and Surgical Procedure

General anesthesia was induced by intraperitoneal administration of ketamine hydrochloride (80 mg/kg) and xylazine hydrochloride (10 mg/kg). Each animal was placed in the supine position, and the anterior cervical region was prepared for surgery. A midline cervical incision was performed, and the strap muscles were separated to expose the thyroid and tracheoesophageal region. The left RLN was carefully identified and dissected.

The left RLN was completely transected. In Group 1, the transected nerve ends were left unrepaired. In Groups 2 and 3, the proximal and distal nerve stumps were approximated and primary end-to-end anastomosis was performed using 8/0 Prolene sutures under 10× microscopic magnification, with care taken to achieve anatomical alignment of the nerve ends without excessive tension.

In Group 3, the freshly prepared donor-derived fibrin matrix was placed directly over and around the completed anastomosis. No fibrin matrix was applied in Groups 1 or 2. Following completion of the experimental procedure, the surgical field was closed in anatomical layers. Postoperative analgesia was provided with subcutaneous meloxicam (1–2 mg/kg) for 5 days. Animals were subsequently returned to the animal care facility and observed during the 3-month recovery period.

### 2.4. Laryngoscopic Evaluation of Vocal Fold Mobility

Laryngoscopic examination was performed before the initial surgical procedure to confirm bilateral vocal fold mobility. Under general anesthesia, the larynx was visualized transorally using a flexible mini-USB endoscope. Following transection of the left RLN, laryngoscopy was repeated to confirm loss of ipsilateral vocal fold movement.

At the end of the 3-month follow-up period, a final laryngoscopic examination was performed before tissue harvesting. Vocal fold mobility was assessed qualitatively by direct visual observation during laryngoscopy and was classified as either mobile or fixed. A vocal fold was considered mobile when visible movement was observed during the examination and fixed when no visible movement was observed throughout the examination. No quantitative grading of movement amplitude, velocity, or glottic opening was performed.

### 2.5. Tissue Harvesting and Histopathological Evaluation

Following the final laryngoscopic examination at 3 months, the animals were re-anesthetized and the cervical surgical field was re-explored. A segment of the left RLN encompassing the previous transection or repair site was excised for histopathological examination. Vocal fold tissue specimens were also harvested to assess denervation-related muscle changes. The animals were subsequently euthanized under anesthesia.

Tissue specimens were fixed in 10% formalin and processed for histopathological examination. RLN specimens were evaluated for inflammation, fascicular organization disorder, vascular proliferation, edema, congestion, foreign-body reaction, and axon count. Histopathological changes were graded semiquantitatively according to their severity as absent, mild, or marked, where applicable. Axon count was evaluated quantitatively. Vocal fold specimens were examined for muscle atrophy. The degree of vocal fold muscle atrophy was recorded according to the histopathological findings and categorized as absent, mild, marked, or absence of identifiable muscle tissue, as applicable.

Histopathological changes were graded semi-quantitatively as absent, mild, or marked according to their extent and intensity. “Absent” was defined as no appreciable morphological alteration, “mild” as focal or limited changes involving only a portion of the examined tissue, and “marked” as prominent and widespread changes involving a substantial part of the examined tissue. All histopathological assessments were performed by a pathologist blinded to the experimental treatment allocation; specimens were identified only by coded group numbers.

### 2.6. Outcome Measures

The primary functional outcome was recovery of left vocal fold mobility at 3 months. Histopathological outcomes included the degree of vocal fold muscle atrophy and changes at the RLN injury/repair site, including inflammation, fascicular organization disorder, vascular proliferation, edema, congestion, foreign-body reaction, and axon count. Functional and histopathological outcomes were compared among the untreated transection, primary microsurgical repair, and fibrin-matrix-supplemented repair groups.

### 2.7. Statistical Analysis

Statistical analyses were performed using IBM SPSS Statistics for Windows, version 29.0 (IBM Corp., Armonk, NY, USA). Categorical variables were summarized as numbers and percentages, whereas ordinal and continuous variables were expressed as median and interquartile range (IQR), as appropriate. Vocal fold mobility was treated as a categorical outcome and compared among the three groups using an exact test appropriate for small sample sizes. Histopathological variables recorded on an ordinal scale were compared among the three groups using the Kruskal–Wallis test. When an overall significant difference was detected, post hoc pairwise comparisons were performed with correction for multiple comparisons. Axon counts were compared among groups using the Kruskal–Wallis test because of the small group sizes and distributional characteristics of the data.

All tests were two-sided, and a *p*-value < 0.05 was considered statistically significant red.

## 3. Results

### 3.1. Experimental Course and Vocal Fold Mobility

No major complication affecting the experimental protocol was observed during surgery or the 3-month follow-up period. At the end of follow-up, none of the animals in Group 1 showed recovery of left vocal fold mobility. Vocal fold mobility was observed in 2 of 9 animals (22.2%) in Group 2 and in 4 of 9 animals (44.4%) in Group 3. Thus, the proportion of animals with recovered vocal fold mobility increased from the untreated transection group to primary repair and was highest following primary repair supplemented with fibrin matrix. However, the overall between-group difference did not reach statistical significance (Fisher–Freeman–Halton exact test, *p* = 0.116) (Table 1). Representative laryngoscopic images of a mobile and a fixed left vocal fold are shown in Figure S1. Corresponding dynamic laryngoscopic recordings are provided as Supplementary Videos S1 and S2.

The absence of major complications affecting the protocol and the observed 0/9, 2/9, and 4/9 mobility pattern were also documented in the original presentation.

### 3.2. Vocal Fold Muscle Atrophy

The distribution of vocal fold muscle atrophy differed significantly among the three groups (*p* < 0.001). All animals in Group 1 demonstrated marked muscle atrophy. In Group 2, mild atrophy was observed in 3/9 animals (33.3%) and marked atrophy in 4/9 (44.4%); in the remaining two specimens (22.2%), no identifiable muscle tissue was present in the examined section. In contrast, 7/9 animals (77.8%) in Group 3 showed no histological evidence of muscle atrophy, while the remaining 2/9 (22.2%) demonstrated only mild atrophy. No marked atrophy was observed in Group 3. Representative histopathological sections illustrate marked vocal fold muscle atrophy in Group 1, mild atrophy in Group 2, and preserved muscle architecture without atrophy in Group 3 (Figure S2A–C).

Post hoc pairwise exact comparisons with Holm correction demonstrated significant differences between Group 1 and Group 2 (*p* = 0.029), between Group 1 and Group 3 (*p* < 0.001), and between Group 2 and Group 3 (*p* = 0.003). These findings indicate substantially better preservation of vocal fold muscle in animals receiving fibrin-matrix-supplemented microsurgical repair.

### 3.3. Histopathological Evaluation of the Recurrent Laryngeal Nerve

Significant between-group differences were observed in inflammation (*p* = 0.003), fascicular organization disorder (*p* < 0.001), edema (*p* = 0.008), and foreign-body reaction (*p* < 0.001). In contrast, vascular proliferation (*p* = 0.188) and congestion (*p* = 0.304) did not differ significantly among the groups (Table 2). The principal global histopathological differences are consistent with those reported in the original study presentation.

Inflammatory scores were higher in both repair groups than in the untreated transection group. Dunn post hoc analysis with Holm correction showed significant differences between Group 1 and Group 2 (*p* = 0.023) and between Group 1 and Group 3 (*p* = 0.004), whereas Groups 2 and 3 did not differ significantly (*p* = 0.504). Representative nerve sections demonstrated marked fascicular organization disorder in Groups 1 and 2, whereas only mild disorganization was observed in the fibrin matrix group (Figure S2D–F).

Fascicular organization was most preserved in the fibrin matrix group. Fascicular organization disorder was significantly lower in Group 3 than in Group 1 (*p* < 0.001) and Group 2 (*p* = 0.030), whereas the difference between Groups 1 and 2 was not significant (*p* = 0.179).

Edema was absent in all nine animals in Group 3. Representative sections showed mild edema in Groups 1 and 2, whereas no edema was evident in the fibrin matrix group (Figure S3A–C). Edema scores were significantly lower in Group 3 than in both Group 1 and Group 2 (Holm-adjusted *p* = 0.022 for both comparisons), while Groups 1 and 2 were identical with respect to the distribution of edema scores (*p* = 1.000).

Foreign-body reaction was more pronounced in the repaired nerves. Compared with Group 1, foreign-body reaction scores were significantly higher in both Group 2 (*p* = 0.004) and Group 3 (*p* < 0.001). No significant difference was observed between the two repair groups (*p* = 0.419). Histologically, no foreign-body reaction was observed in the representative Group 1 specimen, whereas marked foreign-body reactions were evident in representative specimens from both repair groups (Figure S3D–F). Therefore, the increased reactive tissue response was associated with nerve repair in general rather than being demonstrably greater after addition of the fibrin matrix.

### 3.4. Axon Counts

Median axon counts were 111 [105–114] in Group 1, 87 [84–96] in Group 2, and 103 [73–123] in Group 3. The highest median axon count was observed in the untreated transection group; however, the overall difference among the three groups was not statistically significant (*p* = 0.085). Therefore, the present data do not support a significant effect of either primary repair or fibrin matrix supplementation on total axon count at the 3-month time point.

## 4. Discussion

The principal finding of the present study was that supplementation of primary microsurgical RLN repair with a donor-derived fibrin matrix was associated with substantially better preservation of vocal fold muscle and a more favorable histopathological pattern at the repair site. In contrast, recovery of vocal fold mobility showed only a numerical improvement: no recovery was observed after untreated transection, whereas mobility returned in approximately one-fifth of animals after primary repair and in almost half of those receiving fibrin matrix supplementation. This stepwise increase is biologically noteworthy, but the difference did not reach statistical significance and should not be interpreted as evidence of improved functional recovery. In RLN regeneration, anatomical or histological improvement does not necessarily translate into coordinated vocal fold movement because successful recovery also depends on appropriate motor reinnervation. Therefore, the structural findings of reduced muscle atrophy, less fascicular disorganization, and lower edema should be interpreted separately from the functional endpoint.

This structure–function dissociation is well recognized after experimental RLN injury. Hernández-Morato et al. demonstrated reinnervation after RLN transection and repair despite persistent vocal fold immobility, which was attributed to misdirected axonal regeneration and disruption of the normal organization of motor neuron pools [5]. Similarly, Kumai et al. showed electrophysiological and histological evidence of RLN reinnervation through a silicone conduit despite persistent vocal fold immobility [13]. These findings support the interpretation that improved neural regeneration or tissue preservation may occur without restoration of coordinated vocal fold motion.

The preservation of vocal fold muscle observed in our fibrin matrix group may therefore represent one of the most relevant findings of the study. Denervation-induced laryngeal muscle atrophy is a downstream consequence of loss of neural input, and preservation of muscle architecture may indicate at least partial restoration or maintenance of neuromuscular connectivity even when coordinated movement remains incomplete. Similar preservation of laryngeal muscle despite limited recovery of vocal fold movement has been reported with other regenerative strategies. Suzuki et al. observed better preservation of laryngeal muscle and more clearly regenerated myelinated fibers after reconstruction with a PGA–collagen conduit than after direct suturing, despite the absence of vocal fold movement in either group [14]. Yoshimatsu et al. likewise reported greater preservation of the thyroarytenoid muscle and enhanced neural regeneration with a self-assembling peptide hydrogel, although recovery of vocal fold movement remained uncommon [15]. Taken together with the present findings, these studies suggest that preservation of the target laryngeal muscle may be a sensitive indicator of biological benefit after experimental RLN repair, potentially preceding—or occurring independently from—meaningful recovery of vocal fold motion.

A particularly relevant comparison is the experimental study by Şentürk et al., which used a closely related RLN transection model consisting of untreated transection, primary repair, and repair with a PGA-coated conduit in Wistar rats [16]. Vocal fold mobility was proportionally greater following conduit reconstruction, but the difference among groups was not statistically significant; the conduit group nevertheless demonstrated the highest axon counts, leading the authors to suggest that an isolated regenerative environment may improve orientation of regenerating fibers [16]. Our findings extend this concept from an engineered conduit to a blood-derived biological scaffold. Rather than physically bridging a nerve defect, the fibrin matrix in our model was applied around an already completed primary anastomosis. The observed reduction in fascicular organization disorder suggests that modifying the immediate microenvironment of a technically repaired nerve may influence the subsequent organization of healing, even when the primary mechanical reconstruction is identical.

The biological plausibility of this effect is supported by the characteristics of fibrin-based matrices. Fibrin forms a temporary three-dimensional extracellular scaffold that permits cellular migration and can support Schwann cell activity and axonal extension during peripheral nerve healing [6]. Blood-derived fibrin preparations may additionally retain platelets and locally active biological mediators, although the composition varies considerably according to the preparation protocol. In the present experiment, the material was produced using a PRF-based centrifugation method but was not quantitatively characterized for platelet concentration or growth-factor content; it should therefore be regarded as a donor-derived fibrin matrix rather than as a standardized PRF product [12]. This distinction is important when comparing our findings with studies using platelet-rich plasma. In a recent rat RLN model, Senturk et al. found that primary nerve repair combined with PRP resulted in higher electrophysiological amplitudes, increased Schwann cell proliferation and myelin-related staining, and reduced axonal damage compared with untreated injury or repair without PRP [11]. Although PRP and the fibrin matrix used in our study are biologically and technically different preparations, both findings support the broader concept that blood-derived biological adjuncts may improve the microenvironment surrounding an injured and reconstructed RLN.

The lower degree of fascicular organization disorder in the fibrin matrix group may be more relevant to the quality and organization of nerve regeneration than to the absolute number of axons. Importantly, axon counts did not differ significantly among the groups (*p* = 0.085), and the highest median axon count was observed in the untreated transection group rather than in either repair group. Therefore, the axon-count findings should not be interpreted as evidence of enhanced axonal regeneration following fibrin matrix supplementation. Axon number at a single histological level may also be influenced by regenerative sprouting and does not indicate whether the observed axons are appropriately organized, myelinated, electrically functional, or correctly directed toward their original motor targets. Accordingly, the more favorable fascicular organization and preservation of target muscle observed in the fibrin matrix group should be interpreted independently from total axon number. Without electrophysiological testing and detailed quantitative nerve morphometry, the biological significance of these findings remains uncertain.

Edema was reduced in the fibrin matrix group; however, the same pattern was not observed for inflammation or foreign-body reaction. Inflammatory scores were higher in both repair groups than in the untreated transection group, and foreign-body reactions were likewise more pronounced following surgical repair. Importantly, neither inflammation nor foreign-body reaction differed significantly between the primary repair and fibrin-matrix-supplemented repair groups. Therefore, the present findings do not support an anti-inflammatory effect of the fibrin matrix. Rather, the increased inflammatory and foreign-body responses appear to be associated with the repair procedure itself, including suture placement and additional tissue manipulation. In this context, the favorable findings observed with fibrin matrix supplementation should be limited to reduced edema, improved fascicular organization, and better preservation of target muscle, rather than interpreted as evidence of reduced inflammation. Further studies including immunohistochemical characterization of inflammatory-cell populations and temporal assessment of the tissue response would be required to determine whether the observed inflammatory reaction has beneficial, neutral, or detrimental effects on nerve regeneration.

Our findings should also be interpreted in the context of the limitations of the study. First, the relatively small number of animals in each group limited statistical power, particularly for the binary endpoint of vocal fold mobility. The increase from no mobile vocal folds after transection to four of nine after fibrin-supplemented repair is potentially meaningful, but the present experiment was not sufficiently powered to establish this as a definitive functional effect. Second, vocal fold function was categorized as mobile or fixed. Quantitative assessment of movement amplitude, movement velocity, glottic opening, or high-speed video analysis could provide a more sensitive functional endpoint in future experiments. Third, electromyography and nerve conduction studies were not performed. Their inclusion would have been particularly valuable in animals showing preserved muscle architecture without visible vocal fold motion and could help distinguish incomplete reinnervation from functionally ineffective synkinetic reinnervation. An additional limitation is the absence of a sham-operated control group. Although the transection group underwent surgical exposure and nerve transection, it does not allow the effects of surgical manipulation itself to be distinguished from those caused by RLN injury and subsequent repair. Therefore, the potential contribution of operative exposure and tissue handling to the observed histopathological changes cannot be independently assessed.

Several histological limitations should also be acknowledged. Axon counts alone provide limited information regarding regenerative quality, and assessment of axonal diameter, myelin thickness, myelinated fiber density, and distal neuromuscular junction integrity would strengthen future studies. In addition, most tissue variables in the present study were evaluated semiquantitatively, which inevitably provides less resolution than quantitative histomorphometry. Only a single postoperative time point at 3 months was assessed. Therefore, the present study cannot determine how the observed functional and histopathological changes evolved over time or whether the effects associated with fibrin matrix supplementation were transient or sustained. In particular, the temporal profiles of inflammation, edema, fascicular organization, axonal regeneration, and target-muscle preservation could not be evaluated. Serial assessments at multiple postoperative intervals would be necessary to characterize the time course and duration of the fibrin-matrix-associated effects.

Finally, the fibrin preparation itself represents both a practical feature and an important limitation. The matrix was rapidly prepared from donor rat blood using a relatively simple centrifugation protocol, making the approach technically attractive as an adjunct to microsurgical repair. However, platelet concentration, fibrin architecture, growth-factor content, and matrix volume were not standardized quantitatively. Furthermore, because the material was derived from a separate donor animal, the current experiment cannot be directly extrapolated to an autologous clinical preparation. Autologous and donor-derived fibrin-based matrices should not necessarily be considered biologically equivalent. Autologous fibrin scaffolds contain patient-derived platelet- and plasma-derived biomolecules, and their composition and biological activity may vary according to the preparation protocol and patient-specific biological characteristics [17]. In contrast, donor-derived preparations may introduce additional donor-related biological variability. These differences were not evaluated in the present study, and our findings therefore cannot establish equivalence between donor-derived and autologous fibrin matrices. The use of a single donor animal represents an additional limitation. Although this ensured that all animals in the fibrin matrix group received material from the same biological source, it did not allow assessment of potential donor-to-donor variability in fibrin composition or biological activity. Therefore, the reproducibility of the findings across different donors remains to be established. Future studies should therefore characterize the biological composition of the matrix and compare donor-derived and autologous preparations. From a translational perspective, an autologous matrix prepared intraoperatively from the patient’s own blood would be a more clinically relevant strategy and would avoid concerns related to donor-derived biological material.

Despite these limitations, the present model provides a useful proof of concept. A fibrin matrix applied as a biological adjunct to an otherwise conventional microsurgical end-to-end RLN repair was associated with markedly better preservation of the target vocal fold muscle, reduced fascicular disorganization, and less edema. These structural benefits occurred without a statistically significant increase in vocal fold mobility or axon number, emphasizing once again that anatomical regeneration and coordinated laryngeal function are not interchangeable outcomes. The findings therefore support further investigation of fibrin-based adjuncts to RLN reconstruction, particularly in studies combining quantitative histomorphometry with electromyography and more sensitive assessment of vocal fold motion.

## 5. Conclusions

Supplementation of primary microsurgical recurrent laryngeal nerve repair with a donor-derived fibrin matrix was associated with better preservation of vocal fold muscle and more favorable histopathological findings, particularly reduced fascicular disorganization and edema. Although vocal fold mobility was numerically higher in the fibrin matrix group, this difference did not reach statistical significance, and axon counts were comparable among groups. These findings suggest that the fibrin matrix may improve the quality of neural healing and target muscle preservation without necessarily resulting in immediate restoration of coordinated vocal fold motion. Further studies with larger sample sizes, electrophysiological assessment, quantitative histomorphometry, and standardized fibrin preparations are warranted to clarify its potential role as an adjunct to RLN reconstruction.

## Figures and Tables

**Table 1:** Vocal fold mobility and muscle atrophy at 3 months.

Outcome	Group 1: Transection (*n* = 9)	Group 2: Primary Repair (*n* = 9)	Group 3: Repair + Fibrin Matrix (*n* = 9)	*p* Value
**Vocal fold mobility**				0.116
Mobile	0 (0%)	2 (22.2%)	4 (44.4%)	
Fixed	9 (100%)	7 (77.8%)	5 (55.6%)	
**Vocal fold muscle atrophy**				<0.001
Absent	0 (0%)	0 (0%)	7 (77.8%)	
Mild	0 (0%)	3 (33.3%)	2 (22.2%)	
Marked	9 (100%)	4 (44.4%)	0 (0%)	
No identifiable muscle tissue	0 (0%)	2 (22.2%)	0 (0%)	

Data are presented as *n* (%). Overall comparisons were performed using the Fisher–Freeman–Halton exact test. Pairwise post hoc comparisons for vocal fold muscle atrophy were adjusted using the Holm method.

**Table 2:** Histopathological findings of the recurrent laryngeal nerve.

Histopathological Parameter	Group 1	Group 2	Group 3	*p* Value
Inflammation score	0 [0–1]	1 [1,2]	2 [1,2]	0.003
Fascicular organization disorder	2 [2–2]	2 [1,2]	1 [1–1]	<0.001
Vascular proliferation	0 [0–0]	0 [0–1]	0 [0–2]	0.188
Edema	1 [0–1]	1 [0–1]	0 [0–0]	0.008
Congestion	0 [0–0]	1 [0–1]	2 [0–2]	0.304
Foreign-body reaction	0 [0–0]	2 [2–2]	2 [2–2]	<0.001
Axon count	111 [105–114]	87 [84–96]	103 [73–123]	0.085

Data are presented as median [interquartile range]. Semiquantitative histopathological parameters were scored as 0 = absent, 1 = mild, and 2 = marked. Overall comparisons were performed using the Kruskal–Wallis test. Significant overall comparisons were followed by Dunn’s post hoc test with Holm correction.

## Data Availability

The data generated and analyzed during the current study are available from the corresponding author upon reasonable request.

## Supplementary Materials

The following supporting information can be downloaded at https://www.mdpi.com/article/10.3390/biomedicines14102276/s1, Video S1: Representative laryngoscopic recording demonstrating a mobile left vocal fold at the 3-month evaluation; Video S2: Representative laryngoscopic recording demonstrating a fixed left vocal fold at the 3-month evaluation; Figure S1: Representative laryngoscopic recordings demonstrating mobile and fixed vocal folds are provided as Supplementary Videos S1 and S2; Figure S2: Representative histopathological findings of vocal fold muscle atrophy and recurrent laryngeal nerve fascicular organization. (A–C) Vocal fold muscle sections showing marked atrophy in G1 (transection without repair) (A), mild atrophy in G2 (primary micro-surgical repair) (B), and absence of atrophy with preserved muscle architecture in G3 (primary repair + donor-derived fibrin matrix) (C). (D–F) Recurrent laryngeal nerve sections showing marked fascicular organization disorder in G1 (D), mild fascicular organization disorder in G2 (E), and absence of fascicular organization disorder in G3 (F). These panels illustrate the semiquantitative histopathological grading categories used in the study. All sections were stained with hematoxylin and eosin (H&E) and examined at 200× magnification. Scale bar = 100 μm. Abbreviations: G1, transection without repair; G2, primary micro-surgical repair; G3, primary repair + donor-derived fibrin matrix; Figure S3: Representative histopathological findings of edema and foreign-body reaction at the recurrent laryngeal nerve injury/repair site. (A–C) Recurrent laryngeal nerve sections showing mild edema in G1 (transection without repair) (A) and G2 (primary microsurgical repair) (B), and absence of edema in G3 (primary repair + donor-derived fibrin matrix) (C). (D–F) Representative sections showing no foreign-body reaction in G1 (D) and marked foreign-body reaction in G2 (E) and G3 (F). All sections were stained with hematoxylin and eosin (H&E) and examined at 200× magnification. Scale bar = 100 μm. Abbreviations: G1, transection without repair; G2, primary microsurgical repair; G3, primary repair + do-nor-derived fibrin matrix.

## Abbreviations

The following abbreviations are used in this manuscript:

RLN	Recurrent laryngeal nerve
PRF	Platelet-rich fibrin
H&E	Hematoxylin and eosin
IQR	Interquartile range
ARRIVE	Animal Research: Reporting of In Vivo Experiments
